# Frequency and associated symptoms of isthmoceles in women 6 months after caesarean section: a prospective cohort study

**DOI:** 10.1007/s00404-022-06822-8

**Published:** 2022-11-09

**Authors:** P. Gozzi, K. A. Hees, C. Berg, M. David, K.-D. Wernecke, L. Hellmeyer, D. Schlembach

**Affiliations:** 1 Klinik für Gynäkologie, Campus Virchow-Klinikum, Charité Universitätsmedizin Berlin, Berlin, Germany; 2 Klinik für Gynäkologie, Vivantes Klinikum im Friedrichshain, Berlin, Germany; 3 Institut für Biometrie und Klinische Epidemiologie, Charité Universitätsmedizin Berlin, Berlin, Germany; 4 Klinik für Geburtsmedizin, Vivantes Klinikum Neukölln, Berlin, Germany

**Keywords:** Isthmocele, Myometrial niche, Caesarean scar defect, Postpartum

## Abstract

**Purpose:**

The purpose of this study was to determine the frequency of detection of isthmoceles by ultrasound 6 months after caesarean section (CS) and which symptoms associated with isthmocele formation occur after CS. Subsequently, it was determined how often the ultrasound finding “isthmocele” coincided with the presence of complaints.

**Methods:**

A prospective multicentre cohort study was conducted with 546 patients from four obstetric centres in Berlin, who gave birth by primary or secondary CS from October 2019 to June 2020. 461 participants were questioned on symptoms 3 months after CS; 329 participants were included in the final follow-up 6 months after CS. The presence of isthmoceles was determined by transvaginal sonography (TVS) 6 months after CS, while symptoms were identified by questionnaire.

**Results:**

Of the 329 women, 146 (44.4%) displayed an isthmocele in the TVS. There was no statistically significant difference in the manifestation of symptoms between the two groups of women with and without isthmocele; however, when expressed on a scale from 1 to 10 the intensity of both scar pain and lower abdominal pain was significantly higher in the set of women that had shown to have developed an isthmocele (*p* = 0.014 and *p* = 0.031, respectively).

**Conclusion:**

The prevalence of isthmoceles 6 months after CS was 44.4%. Additionally, scar pain and lower abdominal pain were more pronounced when an isthmocele was also observed in the TVS.

**Trial registration:**

Trial registration number DRKS00024977. Date of registration 17.06.2021, retrospectively registered.

## What does this study add to the clinical work

**Table:** 

This ultrasound-based study detected isthmoceles as common complication of CS. Women with isthmoceles had more severe manifestations of scar pain and lower abdominal pain, however there was no significant difference in presence of symptoms compared to women without isthmoceles.	

## Introduction

Whilst the WHO recommends a caesarean section rate of 10% [2], caesarean sections (CS) have seen a gradual increase as chosen modality of birth in western countries [3] in the past decades. In 2020, Germany reported a CS rate of 29.7% out of all hospital births [4]. In connection with this development, there has been a surge in scientific interest in the topic of isthmoceles [5, 6]. As they represent a long-term complication of CS, their occurrence, and in consequence their detection, might therefore have also become more frequent.

Isthmoceles, also referred to as caesarean scar defects or (myometrial) niches, have been defined as indentations of the anterior uterine wall akin to a diverticulum at the site of myometrial scar of minimum 2 mm in depth [7]. The standard diagnostic procedure for identification of isthmoceles is transvaginal sonography (TVS), however, contrast-enhanced sonohysterography has proven to be an at least equally apt alternative method [8].

The detection rate of isthmoceles after CS is reported with differing numbers, the prevalence ranging from 6.9% to 64.5% depending on the study population and the implemented methodology [1, 9–11]. Previous studies have also described a relationship between isthmoceles and the occurrence of symptoms, especially those of postmenstrual spotting [9–12], pelvic pain and dysmenorrhea [9], and intermenstrual bleeding [10].

Due to the inconsistency of the data gathered so far, a prospective clinical study was performed with the aim to determine the frequency of development of a myometrial niche after CS and a potential association of isthmoceles with specific symptoms.

## Materials and methods

With the objective of examining frequency and associated symptoms of caesarean scar defects in women 3 and 6 months after CS a prospective cohort study was performed. The multicentre study was conducted at the Department of Gynaecology and Obstetrics Campus Virchow and Campus Mitte of Charité University Hospital Berlin and at the Vivantes clinics Klinikum im Friedrichshain and Klinikum Neukölln in Berlin, Germany. The study protocol was reviewed and approved by Charité’s Ethics Committee in June 2019 (application number: EA2/069/19).

From October 15th 2019 to June 18th 2020, women who had given birth by CS at the abovementioned clinics were asked to participate in our study. Due to the onset of the COVID-19 pandemic and the subsequent shutdown of clinical research activities in Berlin’s clinics, fewer participants were enrolled in the period from March 16th 2020 to May 6th 2020 as not all recruiting personnel belonged to the staff working on the ward. Patients were asked to participate as of the first postoperative day and written informed consent was provided after thorough briefing of every participant.

Exclusion criteria included age < 18 years and > 40 years, triplet pregnancies, known uterine anomalies and abnormalities of placental implantation such as placenta accreta spectrum or placenta praevia, and whenever instruction on the study was impossible due to language barriers. Further medical information such as baseline data and surgical reports were obtained from the electronic patient database. Participants were contacted either by phone or email 3 months after the CS. They were asked to answer a set of questions on symptoms and possible complaints, which had arisen since the CS. Participants were invited to a medical examination 6 months after the CS, in which a TVS was performed. Simultaneously, the questions from the first interview were repeated with regard to the 3 months prior to the appointment. The numbers below on how many patients experienced certain symptoms are based on participants’ responses on the questionnaires. These were not always filled out exhaustively by all participants, occasionally not responding (or choosing not to respond) to entire questions. Therefore, not all sets of answers always add up to 100% of our entire sample.

**Fig. 1:**
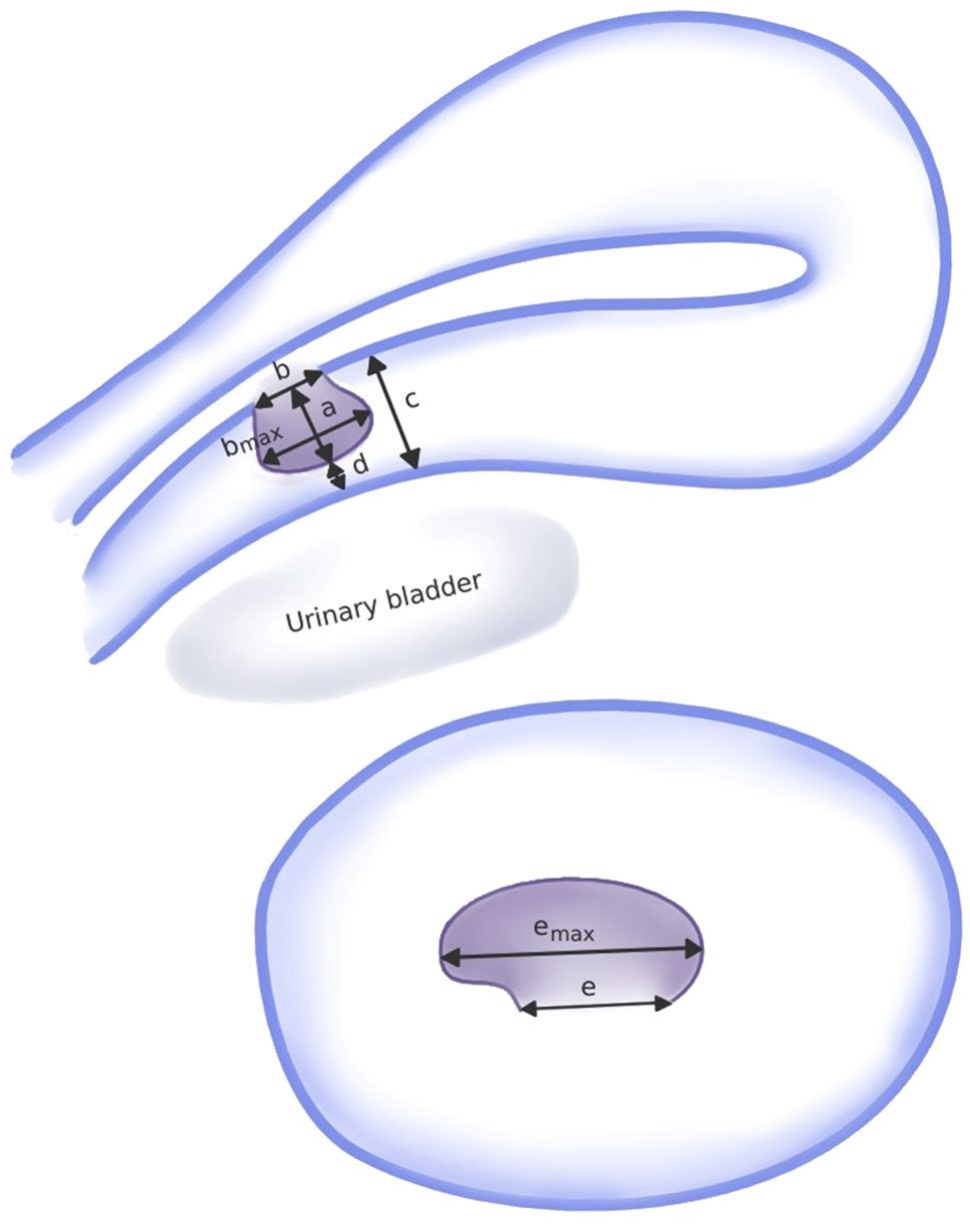
Schematic depiction of an isthmocele; sagittal plane: **a** depth, **b** length, **c** adjacent myometrial thickness (AMT), **d** residual myometrial thickness (RMT); transverse plane: **e** width. Figure modified from Antila-Långsjö et al. [1]

Our definition of a niche is based on Jordans et al.’s [7] description of “an indentation at the site of the CS scar”. Upon identification of a niche, it was measured in depth and length in the sagittal plane and in width in the transverse plane. Additionally, it was screened for the presence of a niche branch and the extents of the adjacent myometrial thickness (AMT) and the residual myometrial thickness (RMT) in front of the niche (Fig. 1) were determined. The sonographic examinations were performed by eight physicians in total, of which five covered 90% of all appointments. Every examiner had at least 1000 prior TVS of experience.

### Statistical analyses

The primary endpoint of the study was to assess the prevalence of isthmoceles 6 months after CS and the association of possible symptoms, with the determination of possible risk factors representing our secondary outcome, which will be discussed in further publications. Under the assumption that 30% of women present with myometrial defects after CS [13] our initial required sample size amounted to 392 participants. This allowed for a power of 90% with two-tailed *p* values of < 0.05 and a drop-out rate of 20%. To ensure meeting the target participant number in case of higher drop-out rates the initial recruitment goal was increased to 500 participants.

Results are expressed as arithmetic mean ± standard deviation or frequencies in percentages, respectively. The data were statistically analysed using statistical software (IBM SPSS Statistics Version 25.0, Version 27.0, and Version 28.0; IBM Corp, Armonk, NY). Initially, differences regarding the development of symptoms within our cohort in relation to the existence of a niche were calculated with Fisher’s exact test. In a second step, the appearance of symptoms was compared between the groups with and without isthmocele along with their intensity measured on a scale from 1 to 10, and statistical differences were determined using the non-parametric exact Mann–Whitney test for independent groups. In addition, a logistic regression model was used to assess the relationship between the development of a niche and certain symptoms. Two-tailed *p* values of < 0.05 were considered statistically significant. Tests of secondary variables were conducted in the area of exploratory data analysis. Therefore, no adjustments for multiple testing have been made.

## Results

**Fig. 2:**
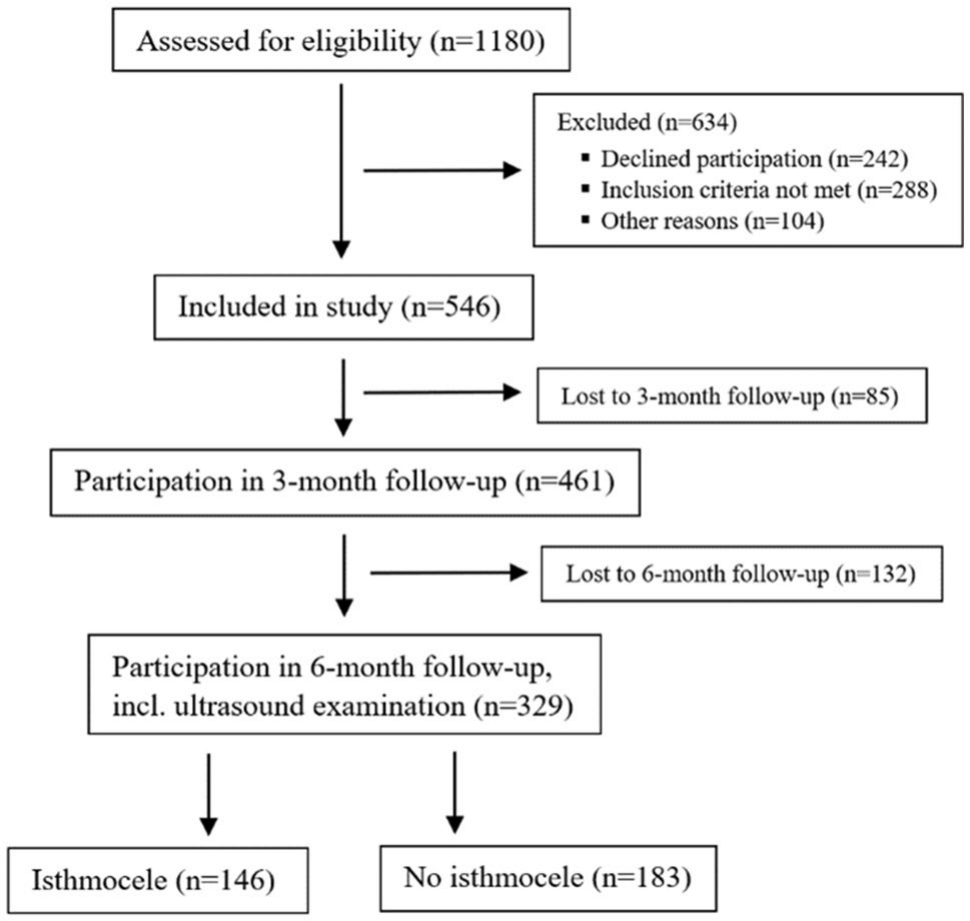
Flow chart of study participants

From October 2019 to June 2020, 546 women gave their written consent to participate in our study. 461 women were reached for the first follow-up after 3 months, and 329 participants presented themselves for the TVS examination on average 6.1 months after CS (Fig. 2).

The participants in the 6-month follow-up had a mean age of 32.83 ± 4.782 years, the average BMI was 26.45 ± 6.05 kg/m^2^. 61 women (18.5%) were diagnosed with gestational diabetes (GDM) during pregnancy, whereas in four cases (1.2%) a type of diabetes mellitus was previously known. For 224 women (68.1%) it was the first CS, 82 women (24.9%) had undergone one previous CS; 22 women (6.7%) had undergone two or more CS in the past. The baseline parameters (at 6-month follow-up) listed by presence or absence of niche are displayed in Table 1. Significant differences between the two groups were found for the number of previous CS (*p* = 0.007) and for parity (*p* = 0.040).

**Table 1:** Baseline characteristics of participants categorised by the presence or absence of niche, data given as mean ± SD at 6 month follow-up

Parameters	Isthmocele	No isthmocele	*P* value
Age (years)	32.97 ± 4.457	32.56 ± 4.978	0.611
Parity	1.66 ± 0.867	1.49 ± 0.818	0.040
History of previous CS	0.52 ± 0.745	0.33 ± 0.664	0.007
BMI (kg/m^2^)	26.78 ± 6.246	26.19 ± 5.99	0.504

When asked 3 months postpartum 200 women (43.4%) stated their menstrual cycle had resumed, whereas for 260 participants (56.4%) their period had not returned. After 6 months 205 women (62.3%) reported on having their period. Though, six months after CS 202 women (61.4%) were still breastfeeding and 70 women (21.3%) were using a hormonal contraceptive method. In 63 cases that meant an oral contraceptive.

### Niche prevalence and anatomical characteristics

Of the 329 women participating in the 6-month clinical follow-up, 146 women (44.4%) displayed a niche at the site of CS in the TVS; in 11 cases (7.5%) the niche showed an additional branch.

The average values for depth, maximum length and maximum width of a niche were 4.4 ± 2.1 mm, 4.6 ± 2.2 mm and 5.2 ± 2.7 mm, respectively. Evaluation of myometrial thickness adjacent to the myometrial niche (AMT) and of the residual myometrium marked from the apex of the niche towards the serosa (RMT) resulted in mean measurements of 10.2 ± 3.7 mm and 5.3 ± 3.2 mm, respectively.

The sonographic characteristics of the uteri of the participants are shown in Table 2 and subdivided by presence or absence of a niche. There was a significant difference between the two groups regarding their endometrial thickness (*p* = 0.007).

**Table 2:** Sonographic characteristics of uteri of participants as measured by TVS at 6 month follow-up; displayed as mean ± SD or in number of participants, respectively

	Isthmocele	No isthmocele	*P* value
Endometrial thickness (mm)	5.3 ± 3.0	4.4 ± 2.7	0.007
Uterine length (mm)	38.4 ± 10.7	37.4 ± 10.7	0.214
Uterine width (mm)	43.8 ± 9.6	42.2 ± 9.5	0.094
Uterine anatomy			0.935
Anteflexed	108 (74%)	138 (75.4%)	
Retroflexed	30 (20.5%)	37 (20.2%)	
Midline	8 (5.5%)	8 (4.4%)	

Upon examination, there were two cases where two isthmoceles could be identified in the same scar. In these cases, we opted to further analyse the larger one. 6 participants had an additional indentation on the outer side of the uterus. One participant displayed a complete dehiscence of the uterine scar.

### Associated symptoms

The frequencies of occurrence of specific symptoms, as declared by the participants who responded to our questionnaire 6 months after CS, are displayed in Table 3. The analysis with regard to presence or absence of a niche is depicted in Table 4.

**Table 3:** Frequency of menstrual disorders and other symptoms at 6-month follow-up

	Post-menstrual spotting	Inter-menstrual bleeding	Dysmenorrhea	Dyspareunia	Vaginal discharge	Lower abdominal pain	Scar pain	Urinary incontinence	Gastro-intestinal disorders
Present	35 (17.1%)	22 (10.7%)	118 (57.6%)	117 (44.8%)	143 (43.5%)	135 (41.0%)	142 (43.2%)	44 (13.4%)	81 (24.6%)
Absent	158 (77.1%)	174 (84.9%)	82 (40.0%)	144 (55.2%)	183 (55.6%)	188 (57.1%)	185 (56.2%)	283 (86.0%)	247 (75.1%)

**Table 4:** Menstrual disorders and other symptoms at 6-month follow-up among women with and without isthmocele

	Isthmocele	No isthmocele	*P* value	OR	95% CI
Postmenstrual spotting	18	17	0.852	1.142	0.549 – 2.377
Intermenstrual bleeding	10	12	0.822	0.853	0.350 – 2.077
Dysmenorrhea	62	56	0.196	1.487	0.843 – 2.622
Dyspareunia	48	69	0.383	0.799	0.488 – 1.309
Vaginal discharge	67	76	0.432	1.214	0.781 – 1.885
Lower abdominal pain	65	70	0.364	1.254	0.804 – 1.955
Scar pain	70	72	0.146	1.394	0.898 – 2.166
Urinary incontinence	24	20	0.147	1.607	0.848 – 3.042
Gastrointestinal disorders	41	40	0.198	1.409	0.852 – 2.332

### Menstrual cycle and disorders

Out of the 205 participants whose period had resumed 6 months after CS, 35 women (17.1%) reported on postmenstrual spotting and 22 women (10.7%) experienced intermenstrual bleeding (Table 3). When asked to estimate the intensity of their menstrual flow on a scale from 1 to 10 an average value of 5.9 ± 2.2 was indicated. 118 women (57.6%) reported on dysmenorrhea (Table 3) with a mean intensity of 4.4 ± 2.3 on a scale from 1 to 10. Evaluating these numbers with regard to the presence or absence of a niche, postmenstrual spotting was present in 18 women without niche and 17 women with niche, intermenstrual bleeding was reported by 12 women without niche and 10 women with niche, and dysmenorrhea was present in 56 women without niche and 62 women with niche (Table 4). There was no statistical difference between the two groups regarding these symptoms (*p* = 0.852; *p* = 0.822; *p* = 0.1966, respectively). Likewise, when comparing the menstrual flow and intensity of dysmenorrhea between the two groups no statistically significant difference was observed (*p* = 0.108; *p* = 0.267, respectively; Table 5).

**Table 5:** Menstrual disorders and other symptoms on a scale from 1 to 10 (mean ± SD) at 6-month follow-up among women with and without isthmocele

	Isthmocele	No isthmocele	*P* value
Menstrual flow	6.08 ± 2.260	5.66 ± 2.048	0.108
Dysmenorrhea	4.86 ± 2.317	4.18 ± 2.208	0.267
Dyspareunia	4.83 ± 3.097	4.94 ± 2.479	0.583
Vaginal discharge	3.57 ± 2.190	3.14 ± 2.296	0.134
Lower abdominal pain	4.22 ± 2.348	3.20 ± 1.846	0.014
Scar pain	3.41 ± 2.116	2.63 ± 1.648	0.031
Urinary incontinence	2.83 ± 1.880	2.85 ± 1.531	0.698
Gastrointestinal disorders	4.37 ± 2.615	4.35 ± 2.966	0.855

### Other symptoms

Additionally, women were asked whether they suffered from dyspareunia, vaginal discharge, lower abdominal pain, scar pain, urinary incontinence and gastrointestinal disorders, under which both constipation and diarrhoea were summarised. 261 of 329 women reported on having resumed sexual intercourse 6 months after CS, therefore, only those participants were asked on dyspareunic complaints. Similarly, only women whose period had resumed were asked on menstrual disorders. The rates at which the aforementioned symptoms occurred 6 months after CS can be abstracted from Table 3. When looking at these frequencies separately within the groups of women with and without isthmocele there was no statistically significant difference in the manifestation of both menstrual disorders and other symptoms (Table 4).

### Severity of symptoms

When comparing the intensity of the abovementioned symptoms in the groups with and without isthmocele, measuring intensity on an ordinal scale from 1 = very low to 10 = highest imaginable, it resulted that both lower abdominal pain and scar pain were significantly more pronounced in women with isthmocele (*p* = 0.014; *p* = 0.031, respectively). On average, women with isthmocele stated a severity of lower abdominal pain of 4.22 ± 2.348 and scar pain of 3.41 ± 2.116, whereas participants who did not display a niche in the TVS reported values of 3.20 ± 1.846 and 2.63 ± 1.648, respectively. Such relationships could not be found for any of the other examined symptoms in regards to their severity (Table 5). Furthermore, the results of univariate logistic regression calculations shown in Table 6 elucidate that the risk of having developed an isthmocele increases for patients with lower abdominal pain or scar pain by about 25% per 1-point increase on the severity scale from 1 to 10 (at 5 additional severity points the risk is threefold).

**Table 6:** Univariate logistic regression analyses on severity of lower abdominal pain and severity of scar pain (indicated on a scale from 1 to 10) in relation to the dependent variable “Isthmocele” (yes/no) at 6 month follow-up

		Coefficient b	*P* value	OR = e^b^	95% CI for OR	
					Lower	Upper
Variable 1	Severity of lower abdominal pain	0.229	0.007	1.257	1.064	1.486
Variable 2	Severity of scar pain	0.223	0.017	1.250	1.041	1.501

## Discussion

### Main findings

In our study cohort, the isthmocele prevalence established by TVS in women who had undergone CS 6 months prior was 44.4%. These findings are comparable to results of other prospective cohort studies, in particular to van der Voet et al.’s study [11], which reported a rate of 49.6% using TVS. Other studies presented prevalence numbers of 45.6% [8], 56% [10] and 69% [14], however, the results provided by Antila-Långsjö et al. and Bij de Vaate et al. were collected by means of saline-contrast (SIS) or gel instillation sonohysterography (GIS), respectively. Other authors published much lower prevalence numbers [9, 15, 16] in TVS-guided examinations, indicating that SIS and GIS may ease the detection of isthmoceles.

Important distinctions between the studies are the point in time at which the clinical exams were carried out (e.g. Dosedla et al. chose 6 weeks and 18 months postpartum [16], whereas other authors examined during time periods of 6–12 weeks [11], 6–9 months [14] or 6–12 months postpartum [10] and thus allowed a more heterogeneous time frame) and the individual definitions of a niche. Whilst ours was based on Jordans et al.’s Delphi study [7] due to their comprehensiveness, Dosedla et al. [16] referred to “severe scar defects” based on the thickness of the scar and the adjacent myometrium. Other studies put more emphasis on the size of the niche [9], using those measurements to detect a relationship with associated symptoms.

This study could not establish isthmoceles’ role in potentially causing symptoms as none of the examined menstrual disorders or other symptoms correlated in a statistically significant manner. This stands in contrast to other prospective studies, where it was shown that in particular menstrual disorders such as intermenstrual bleeding [10], dysmenorrhea [9], postmenstrual spotting [9–12] and chronic pelvic pain [9] were connected to the presence of an isthmocele. A reason for this could be the time of recruitment of participants: as opposed to our study, where women were asked to participate within the first days after CS whilst still in-patient, other studies [9, 15] signed up participants within the scope of other unrelated gynaecological assessments at a later moment in time.

To the best of our knowledge, our study is singular in regards to the examination of both occurrence of symptoms and the intensity of their manifestation. It demonstrated that both lower abdominal pain and scar pain were significantly more severe when in the presence of a niche compared to women with no niche. Hence, we concluded that each additional point on the symptom severity scale for these two symptoms increases the patient’s risk of having acquired a niche by 25%.

In our cohort, dysmenorrhea and dyspareunia occurred in 57.6% and 44.8% of women, respectively. Similarly, Wang et al. observed dysmenorrhea in 53% of their patients, whereas 18.3% suffered from dyspareunia [9]. In contrast to our study population, they excluded patients who suffered from other uterine pathologies from participating, and the time of questioning was not uniform for all participants. Dosedla et al. probed frequencies of dysmenorrhea and dyspareunia at 9% and 21.5%, respectively [16]. Their study population of primiparous women was questioned 18 months after CS. A possible explanation for the discrepancy in our frequencies might be the earlier timing of examination and questioning, when healing and its pain manifestation might still be in progress. In addition, this first postpartum period is influenced by factors such as lactation and hormonal changes (i.e. lower oestrogen levels), which can explain higher rates of dyspareunia [17]. Our study design did not exclude women with other previously existing gynaecological diagnoses, which may, however, constitute a cause of pain independently from the birth experience. Another consideration concerns the fact that the majority of our patients were recruited in two major Berlin clinics with a large clientele of migratory background. Both expression and evaluation of pain have been shown to be socio-culturally conditioned [18] and the postpartum setting is an area where disparities in pain reporting (and in pain management) amongst different ethnic groups exist [19] and should be recognised.

### Strengths and limitations

Our study is one of the few prospective cohort studies carried out in a multicentre setting. This enabled us to increase our sample size by recruiting parallelly in 4 different obstetric centres and thus yield more representative data. Additionally, having participants who had been cared for in different obstetric centres by different physicians allowed us to analyse a more heterogeneous population in terms of surgical practice and aftercare, thereby reducing the possibility of skewing the results towards a less generalisable cohort.

To reduce any possible selection bias in the recruitment process, every woman who had undergone CS in the previous days was asked to participate regardless of indication, obstetric history or mode of CS (elective vs. emergency). Women with placental abnormalities were excluded to reduce the possibility of confounding, although as of now we have no certainty of this connection.

To avoid a potential bias in reporting during the 6-month follow-up, the participants were asked to respond to the questionnaire before the clinical examination, so not to shift their recalling dependent upon whether a niche was found or not. However, a symptom questionnaire always relies on the participants' subjective experience and thereby carries a limitation towards its validity.

Recruiting was performed by 3 members of our study team, who differed in their professional position (2 residents, 1 doctoral student) and also in regularity of recruiting. Though this was not deliberately implemented, it potentially implies a limitation to our study cohort.

The onset of the COVID-19 pandemic constituted another limiting factor to our scientific pursuit. The first restrictions put in place in Germany in March 2020 temporarily stopped our up to then daily recruitment activities. Similarly, a considerable number of women who had initially agreed to participate dropped out after the 3-month follow-up (performed as phone call or email), often quoting worries about possible health risks as their reason for not wanting to attend an elective examination in one of our clinics for the primary scope of research.

### Clinical implications and relevance

Several hypotheses have been presented on the aetiology of isthmoceles [20]: very low uterine incisions, such as performed in CS during contractions after cervical effacement [21], can hinder wound healing due to the presence of mucus-producing glands; different suturing techniques have been proposed as impact factors on uterine healing and therefore on development of isthmoceles, however, Roberge et al. could not confirm this in their systematic review [22]; adhesions as consequence of past surgeries have been suggested to cause traction on the uterine wall thereby interfering with uterine healing. Additionally, several studies have looked at patient-related factors such as previous caesarean deliveries [1, 15], maternal body mass index and gestational diabetes [1].

Whilst our study examined the clinical implications of isthmoceles in terms of symptomatic disturbances, other long-term complications have been described, as well. With an incidence of 1:1800 to 1:2216 caesarean scar ectopic pregnancies are a rare event [23], however, one that carries a high risk of morbidity for the affected woman. A recent case report described the case of a woman with a history of 3 CS afflicted by symptomatic endometriosis that had developed within an isthmocele [24]. And Calzolari et al. [25] reported on a subgroup of infertile patients where isthmoceles were identified as primary cause of infertility and additionally showed that isthmoplasty could restore fertility in 56% of cases.

Although the data so far is rather suggestive of clinical ramifications and not yet conclusive, physicians should not neglect isthmoceles as potential cause of both gynaecological symptoms and of long-term complications when attending to women with a certain obstetric history.

## Conclusion

This multicentre prospective study indicates isthmoceles to be a common long-term consequence of caesarean deliveries. No statistically significant link between the presence of an isthmocele and clinical symptoms could be established in this cohort; however, it was demonstrated that increased intensity of both lower abdominal pain and scar pain manifesting in women after CS raises the risk of an isthmocele causing the disturbances. Overall, isthmoceles represent a differential diagnosis in patients with history of CS and further research into the matter is advised.
